# State-Issued Identification Cards Reveal Patterns in Adult Weight Status

**DOI:** 10.3390/ijerph120606388

**Published:** 2015-06-08

**Authors:** Daniel S. Morris, Eric C. Main, Jenine K. Harris, Abraham Moland, Curtis Cude

**Affiliations:** 1 School of Community Health, Portland State University, Portland, OR 97201, USA; E-Mail: abe.moland@gmail.com; 2 Oregon Health Authority Public Health Division, Portland, OR 97232, USA; E-Mails: eric.c.main@state.or.us (E.C.M.); Curtis.g.cude@state.or.us (C.C.); 3 George Warren Brown School of Social Work, Washington University in St. Louis, MO 63119, USA; E-Mail: harrisj@wustl.edu

**Keywords:** BMI (Body Mass Index), obesity, surveillance, driver card, driver license, identification card

## Abstract

Background: State-issued identification cards are a promising data source for neighborhood-level obesity estimates. Methods: We used information from three million Oregon state-issued identification cards to compute age-adjusted estimates of average adult body mass index (BMI) for each census tract in the state. We used multivariate linear regression to identify associations between weight status and population characteristics, food access, commuting behavior, and geography. Results: Together, home values, education, race, ethnicity, car commuting, and rural-urban commuting area (RUCA) explained 86% of the variation in BMI among tracts. BMI was lower in areas with higher home values and greater educational attainment, and higher in areas with more workers commuting by car. Discussion: Our findings are consistent with other research on socioeconomic disparities in obesity. This demonstrates state-issued identification cards are a promising data source for BMI surveillance and may offer new insight into the association between weight status and economic and environmental factors. Public health agencies should explore options for developing their own obesity estimates from identification card data.

## 1. Introduction

Obesity prevention is a top public health priority; in 2010, one-third of adults and 17% of children and adolescents in the United States were overweight by an unhealthy amount [1]. Obesity, which is strongly associated with a host of chronic health problems, appears to trigger changes in the body’s physiology that make losing weight difficult, accentuating the importance of obesity prevention [2,3]. For most of the U.S., weight status estimates are available at the county level [4]. These data are valuable for monitoring trends, but seldom provide enough detail to focus local efforts. Data from state-issued driver licenses and identification cards (Driver and Motor Vehicle Services Division (DMV) data) may fill the gap.

Morris *et al.* demonstrated that DMV data are reliable for tracking population weight status.^5^ Analysis of Oregon DMV data shows men underreport their weight by 2% and women underreport by 5%, on average, compared to telephone surveys. This bias appears consistent over time, however, thus the data remain useful for tracking [5,6]. Because every record includes an address, DMV data can be used to generate estimates for very small areas, like neighborhoods. As nearly every adult resident is included in the dataset, DMV data can inform community efforts throughout the state. These small-area estimates may offer additional insight into potentially changeable neighborhood influences on obesity, such as the food environment and neighborhood walkability.

Because DMV records are a novel data source for public health tracking, research is needed to determine whether estimates based on these data can be trusted. If BMI estimates from DMV records are reliably biased, patterns should match up with demographic and community factors known to be associated with obesity. 

Like other public health threats, obesity prevalence is greater among groups with less education and lower incomes [7,8,9]. In 2009, 28% of Oregon adults who did not finish high school were obese, compared to only 18% of college graduates. Adults with household incomes over $50,000 had the lowest obesity rates [10]. In addition, Oregon BRFSS data show disparities in adult obesity by race and ethnicity. Obesity rates in Oregon are higher among residents with Latino and American Indian ancestry, and lower among people who trace their roots to Asia or Pacific Islands [10]. If DMV data are consistently biased, we expect small-area weight status estimates will correlate with demographics, as seen in other studies [11,12]. 

Weight loss or gain is largely a function of the foods people eat and the energy they expend, but genetic and environmental factors have a strong influence as well. Millions of Americans live in food deserts, where access to fresh, healthy, and affordable food is severely limited [13], or food swamps, where healthy options are overwhelmed by unhealthy ones [14]. The food environment is generally worse in low-income areas [15,16], which complicates studies on the links between food environments and weight status, though some studies have found associations [17,18]. The research is clearer that sidewalks, cross-walks, parks and trails, bike lanes, and urban design can all promote physical activity [19]. Recent studies find obesity prevalence is higher in areas that are less walkable [18,20,21]. Designing and creating neighborhoods and communities that make physical activity attractive and convenient produce a variety of co-benefits, including physical health impacts, such as influences on chronic diseases and obesity [19]. Most of the population growth in the U.S. since the 1950s has been in suburban areas, away from places where jobs and services are concentrated [22]. Obesity prevalence tends to be higher outside of metropolitan areas [23,24,25,26,27,28]. Living far from critical destinations like work, grocery stores, and medical care providers can mean a lot of driving instead of a short walk. For every hour per day people spend in cars, their risk of obesity goes up by 6%; for every kilometer they walk, their risk goes down 5% [29]. Associations between DMV estimates and measures of the food environment and built environment would provide additional evidence in support of using DMV data to inform public health work.

## 2. Experimental Section

ESRI ArcGIS 10.0 was used to geocode DMV records and SPSS 19.0 was used for analysis. Data for this study came from 3 million Oregon driver licenses and identification cards issued to adults ages 18–84 years between 2005 and 2012. The Driver and Motor Vehicle Services Division (DMV) of the Oregon Department of Transportation provided data for this study. Obtaining the data was not difficult, because Oregon’s DMV recognized that the state public health agency had a legitimate claim to use the data for public health surveillance. However, since DMV did not maintain a codebook for their data set, it took several conversations with DMV staff to identify all the right variables to use for data cleaning and analysis. 

Accounting for multiple cardholders per residence, and also for numerous cardholders at a given site per multiunit housing, about 1.7 million addresses were available for geocoding. Nearly 1.5 million addresses were geocoded; 90% of the addresses were successfully geocoded to a tax lot and 9% to a street. Based on the geocoding, we assigned each address to a census tract. Most of the remaining records were geocoded to city or postal code area centers and not used for this study. 

Body mass index (BMI, in units of kg/m^2^) is the standard measure used to track the weight status of populations. General BMI classifications do not always accurately reflect an individual’s body composition (e.g., body builders have high BMI scores because of their muscle mass, but do not have excess body fat, like most people with a similar BMI). However, BMI remains the best measure for tracking population trends, as it correlates strongly with clinical assessments and health outcomes and is easily computed [22]. We computed BMI for each record and excluded outliers in height (less than four feet or greater than seven feet), weight (less than 50 pounds or greater than 600 pounds) and BMI (less than 14 kg/m^2^ or greater than 68 kg/m^2^). Only 0.024% of the records were outliers, so excluding them was unlikely to affect results. For each census tract in the state, we computed average BMI for all adults and for men and women separately. For analysis, these estimates were age-adjusted using the 2010 U.S. standard population. The Oregon DMV does not collect data on race or ethnicity.

**Figure 1:**
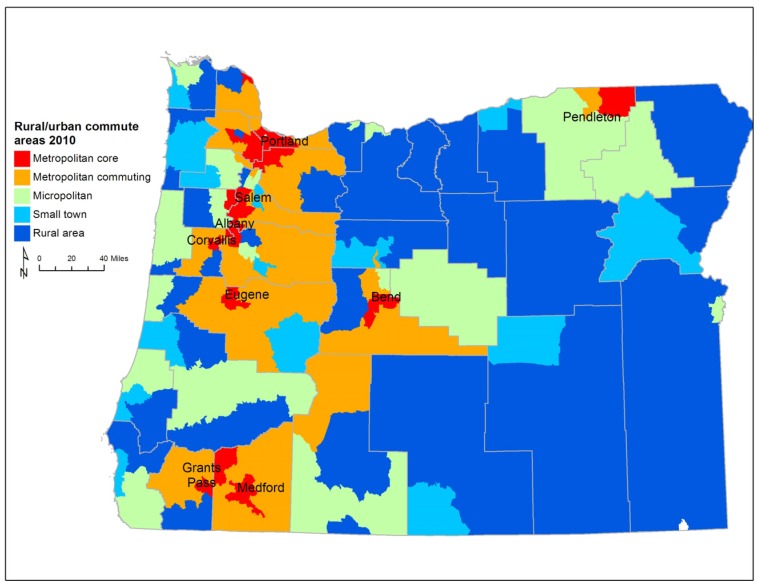
Rural-urban commuting areas in Oregon.

There are several different schemes for defining urban and rural areas throughout the United States [30,31]. For our study we use Rural-Urban Commuting Areas codes (RUCA) to delineate areas for analysis [32] (Figure 1). Since commuting to work plays an important role in defining a person’s lifestyle, RUCAs, which are based on commuting behavior, are a good classification scheme to use for analysis of average weight status. RUCA designations are available at the Census tract level. RUCAs define metropolitan core, metropolitan commuting, micropolitan, small town, and rural areas. In metropolitan core areas the primary commuting flow is within the urbanized area. Metropolitan commuting areas are adjacent to larger cities, with a large share of workers commuting into the city. These areas are typically thought of as suburbs. Micropolitan areas are centered on urban clusters of 10,000 to 49,999 people; these places are sometimes referred to as large rural towns. Small town areas have urban clusters of 2500 to 9999 people and rural areas have fewer than 2,500 people clustered in one place. 

Data on population characteristics came from the American Community Survey (ACS), an ongoing survey conducted by the Census Bureau since 2005. We used five-year ACS data (2006–2010) for the most stable and reliable Census tract level estimates. We analyzed ACS data on race, ethnicity, education, median home value, income, and commuting to work. 

Estimates of the average intersection density (a simple measure of neighborhood walkability) were calculated from the 2010 Census Topologically Integrated Geographic Encoding and Referencing (TIGER) street network. Excluding intersections that led to cul-de-sacs, we counted the intersections within a one-square mile grid and used zonal statistics to create average intersection density per square mile for each census tract and block group. Intersection density is a widely used measure that is feasible to calculate for large areas [33]. 

Measures of proximity to various businesses were calculated using 2010 data from the Oregon Employment Department. Using North American Industry Classification System (NAICS) codes, we classified businesses as fast food restaurants, restaurants (including fast food), convenience stores, grocery stores or produce stands. We supplemented the produce stand list from a 2013 directory maintained by the Oregon Farmers’ Markets Association [34]. From each geocoded DMV address, we computed proximity along the street network to the nearest business of each type. We created measures of average proximity (in miles) to each type of business for each geographic area, along with the percent of residents who live within a mile of each type of business. This is a more meaningful measure than one based on the density of businesses in an area, which is skewed in large rural areas that include towns where the population is concentrated. Proximity measures for assessing the food environment are recommended by the Centers for Disease Control and Prevention [35]. 

This study used an ecological, cross-sectional design. We computed summary statistics by RUCA type, weighting estimates by population counts where appropriate. We then fit linear regression models to the dependent variable of age-adjusted mean BMI at the tract level. Out of a total of 834 Census tracts, nine tracts with fewer than 100 people, according to the 2010 Census, were excluded from analysis, as were three additional tracts with missing home values in the ACS data. Variables were added to the model one at a time and retained if they improved model fit (as indicated by reduction in AIC scores or a statistically significant increase in adjusted R^2^) and beta coefficients were statistically significantly distinct from zero. We tested different classifications of the same constructs and kept the one that performed best. For example, models that described educational attainment using multiple categories performed better than models using a single “years of education” variable. The model was developed for the dependent variable of age-adjusted mean BMI for all adults, then for men and women separately. The tract-level models for men and women used sex-specific educational attainment rates. 

## 3. Results and Discussion

Sixty percent of Oregon’s population resides in metropolitan core areas. These areas have the highest educational attainment rates and the largest non-white populations. Metropolitan core areas contain both the tracts with the highest median incomes and the lowest median incomes in the state. They also contain the tracts with the lowest and highest median age. Home values are lower and vary less across micropolitan and metropolitan commuting areas. Statewide, average age-adjusted BMI was 25.7 kg/m^2^ for women and 27.3 kg/m^2^ for men. Not controlling for other factors, BMI was highest in micropolitan and small town areas and lowest in metropolitan core areas (Table 1).

Metropolitan core areas and rural areas had the lowest percent of workers commuting by car. In rural areas 13.7% of people worked at home, while in metropolitan core areas 8.9% commuted by bicycle or public transit. Walking to work was most common in small town and rural areas in Oregon (5.8%); micropolitan and metropolitan commuting areas had the smallest percent of workers commuting on foot (3.5%). 

People in metropolitan areas in Oregon are less likely to live in a USDA-designated food desert. About 85% of households in metropolitan core areas are within a mile of a restaurant and most are within a mile of a supermarket. Only about one in five houses in metropolitan commuting and rural areas are within one mile of a supermarket, but about twice as many are within a mile of a restaurant. People in rural and micropolitan areas were more likely to live in a food desert, but even so, a quarter of the population in rural areas lives within one mile of a supermarket. Intersection density, a measure of walkability, was twice as high in metropolitan core areas than in micropolitan areas. 

**Figure 2:**
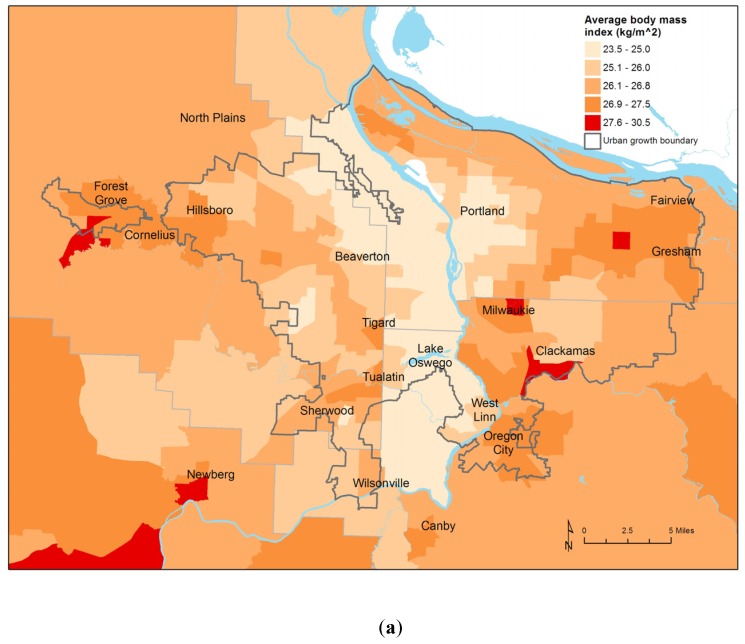

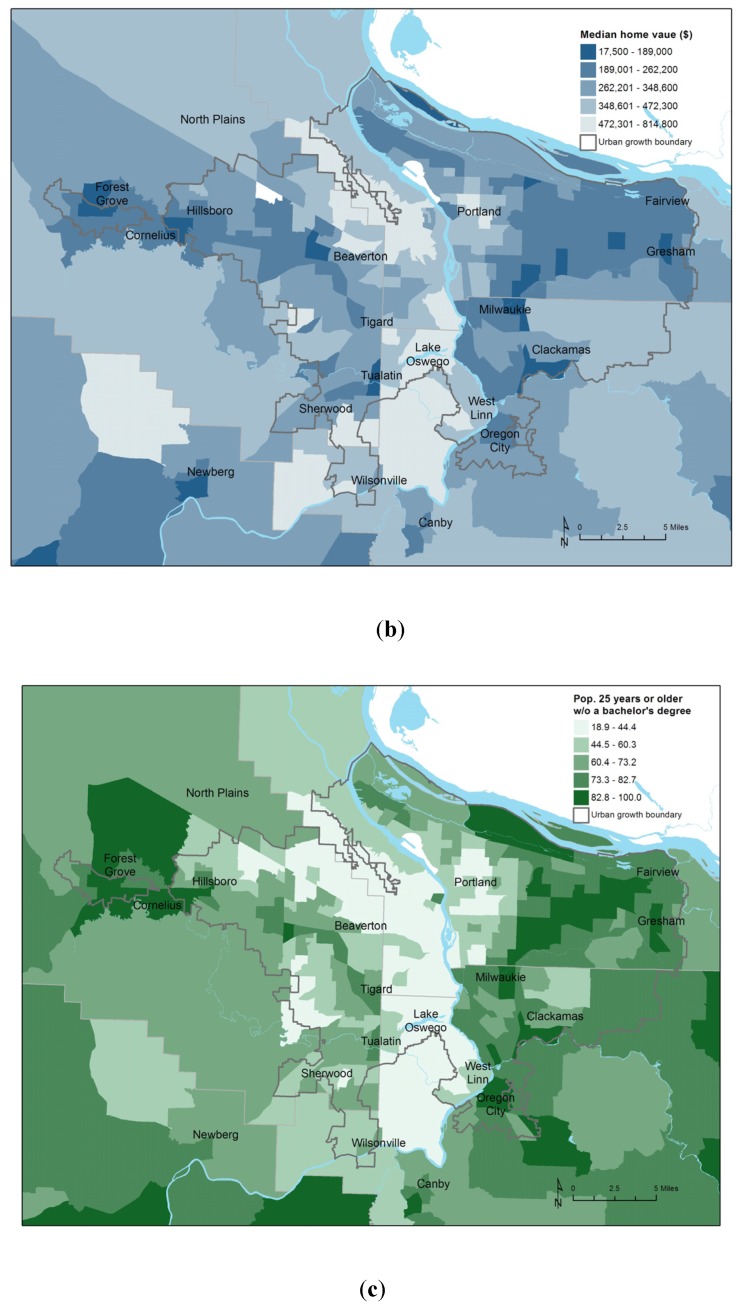
(**a**) Average BMI by Census tract, Portland metropolitan area; (**b**) Median home value; (**c**) Percent of adults 25 years and older without a college diploma.

The statewide regression models explained most of the observed variation in BMI (R^2^ = 0.85 for women, R^2^ = 0.81 for men) (Table 2). Median home value was the single strongest predictor of BMI, with the effect about three times as strong among women as men. As expected, BMI estimates were higher in less affluent areas. For every $100,000 median home value increased, women’s average BMI was 0.45 kg/m^2^ lower. These results are consistent with Drewnoski, Rehm, and Solet’s study on obesity in the Seattle, Washington area [11]. We found home value explained more of the variance in BMI than did measures of income and poverty, which were not retained in the final models. BMI was also higher in areas where educational attainment was lower; this effect was more pronounced among women. The relationship between BMI and socioeconomic status was striking. In the Portland area, average BMI, median home value, and educational attainment show very similar patterns. (Figure 2). 

**Table 1:** Tract level statistics by RUCA category.

Census Tract Characteristic	Metropolitan Area Core (N = 508)	Metropolitan Area Commuting (N = 99)	Micropolitan Area (N = 138)	Small Town (N = 32)	Rural Area (N = 45)
2011 Population	2,455,876	419,537	632,057	143,854	150,661
Education, % of adults with					
No high school diploma	10.2	10.3	13.7	14.5	14.2
High school diploma	22.0	29.7	30.7	32.4	33.2
Some college	33.4	38.7	36.5	35.1	35.3
4-year college degree	21.6	14.0	12.5	11.8	11.5
Graduate degree	12.8	7.3	6.7	6.1	5.8
Race/ethnicity					
% Asian	5.6	1.0	1.2	0.8	0.8
% Black	2.3	0.3	0.5	0.5	0.6
% Native American	1.3	1.0	2.0	1.9	4.3
% Hispanic	12.0	6.2	13.4	11.1	9.7
% White	82.8	93.1	88.2	90.7	89.0
Median age range	19–61	27–57	22–69	32–54	20–56
Median home value range ($ thousands)	17–815	162–546	89–419	115–422	97–419
Median household income range ($ thousands)	10–147	32–96	20–70	23–59	25–64
Age-adjusted mean BMI, all adults (Standard deviation)	26.1 (1.1)	26.7 (0.7)	27.1 (0.6)	27.1 (0.6)	26.8 (0.9)
Females only (Standard deviation)	25.2 (1.4)	25.7 (0.8)	26.4 (0.8)	26.4 (0.8)	25.9 (1.0)
Males only (Standard deviation)	27.0 (0.9)	27.6 (0.5)	27.9 (0.6)	27.8 (0.4)	27.6 (0.8)
% population living in USDA defined food desert	8.8	6.0	20.4	11.5	32.2
Percent of households within 1 mile					
Convenience store	65.3	24.1	41.2	39.6	12.8
Fast food restaurant	74.6	27.0	52.7	49.2	17.7
Any restaurant	82.0	33.7	62.7	62.8	34.3
Supermarket	56.0	22.4	43.3	46.9	25.7
Produce stand	23.9	12.7	21.0	27.8	11.5
Workers commuting by car (%)	80.3	87.7	88.3	86.5	77.6
Intersections per square mile	100.8	24.2	50.1	36.4	9.1

**Table 2:** Linear regression results on age-adjusted mean BMI, statewide analysis by census tract (N = 822).

Census Tract Characteristic	All Adults	Women	Men
Constant	**23.727**	**23.131**	**24.154**
Median Home Value (per $100,000)	**−0.29**	**−0.45**	**−0.14**
Education			
% with no high school diploma	**0.030**	**0.044**	**0.013**
% with high school diploma	**0.035**	**0.036**	**0.033**
% with some college	**0.030**	**0.030**	**0.027**
% with a bachelor or graduate degree (reference)	**--**	**--**	**--**
Race			
% White (reference)	**--**	**--**	**--**
% African American	**−**0.003	**0.010**	**−0.015**
% Native American	**0.032**	**0.039**	**0.025**
% Asian or Pacific Islander	**−0.011**	**−0.014**	**−0.012**
% Other	0.006	**0.012**	0.000
% Hispanic ethnicity	0.004	0.004	0.003
% of Workers commuting by car	**0.014**	**0.013**	**0.019**
Rural urban commuting area			
Metropolitan core (reference)	**--**	**--**	**--**
Metro commuting	**−**0.006	**−**0.071	0.010
Micropolitan	**0.153**	**0.123**	**0.164**
Small town	0.000	**−0**.067	0.038
Rural	**−0.183**	**−0.387**	0.001
Adj. R^2^	0.86	0.85	0.81
**Bold** indicates *p* < .05			

BMIs in micropolitan areas were statistically significantly higher than in metropolitan core areas, while rural areas had significantly lower mean BMIs than metropolitan areas for all adults and women. Metro commuting and small town tracts were not statistically significantly different from metropolitan cores. These results challenge previous research, which found higher obesity rates in rural areas, but are not necessarily inconsistent. Tract-level BMI data enabled us to use a more detailed urban/rural classification scheme than other studies, and as a result we may have revealed differences between metropolitan, micropolitan, small town and rural areas that were previously hidden. Other studies that identified disparities in health risks and outcomes between urban and rural areas used simpler classification schemes to compare urban and rural areas at the county level, thus the resolution of their analysis was not as precise as ours [23,24,25,26,27,28]. 

Race and ethnicity explained a small but statistically significant portion of the variance in BMI, with BMI consistently higher in areas with larger Native American populations and lower in areas with larger Asian populations. These finding are consistent with Oregon BRFSS data on obesity disparities by race. In contrast to the BRFSS, Hispanic ethnicity was not significantly associated with tract-level BMI estimates. It could be that the association was obscured after controlling for education and home values.

For every 10% of the population that commutes to work by car, mean BMI was higher by about 0.1 kg/m^2^. After controlling for other factors, intersection density and measures of proximity to businesses did not account for a meaningful amount of variation in BMI in our models. Other studies have found similar results [36,37,38]. These effects may be subsumed within the RUCA variable. The community design factors were not retained in the final models. 

Even though reporting bias is evident in the DMV data, the patterns observed in our statewide analysis were still consistent with other studies. We conclude DMV data are a powerful tool to guide public health obesity prevention work, and strongly encourage other public health practitioners in other states to collect and analyze DMV data. Not every state collects both height and weight information, but enough do to warrant building a nationwide dataset of tract-level BMI estimates from DMV data [6]. The Federal Driver’s Privacy Protection Act says personal information on DMV records may be disclosed “For use by any government agency, including any court or law enforcement agency, in carrying out its functions, or any private person or entity acting on behalf of a Federal, State, or local agency in carrying out its functions” [39]. Public health surveillance is a core government function, so DMV data should be accessible to public health agencies. Agencies should highlight the safeguards they use to protect confidential health data to demonstrate they will be good stewards of DMV data.

There are many factors related to a person’s weight status, but measures of affluence and education were the strongest predictors of average weight status at the population level. Almost all of the observed variation in average BMI for Census tracts in Oregon could be explained with a few variables relating to socioeconomic status, location of residence, and commuting behavior. Our study expanded on other research focused on metropolitan areas [17,40], demonstrating that associations are present across the entire state. 

Population-level associations between socioeconomic conditions and BMI were stronger for women than for men. This result may reflect economic realities in America, where women and people of color earn lower wages for full-time work and are more likely than men to live in poverty [41]. Interventions that improve social and economic conditions will ultimately have the biggest impact on population health [42]. 

We did not find meaningful associations between weight status and intersection density or average proximity to restaurants or groceries. This result is not a judgment on the efficacy of interventions that make it easier for people to walk or bike safely, or to access fresh fruits and vegetables It could very well be that the effects of these environmental factors are captured by the median home prices or RUCA variables. Also, associations may simply be obscured because our statewide measures did not describe neighborhood environments in enough detail. We did not do any ground truthing or other validity testing of the food environment measures. Examining the influence of community factors within a smaller study area, and measuring the environment in more detail, may produce different results. Based on our findings, additional research in micropolitan areas seems especially warranted. 

Racial disparities are evident in our study but perhaps less pronounced than they would be elsewhere because Oregon’s population is predominantly white. Higher BMIs in areas with larger Native American populations highlight disparities on and around tribal lands. Replicating this study in states with more diverse populations may yield different results. 

### Strengths and Limitations

To our knowledge, this is the first statewide study on disparities in weight status to be conducted at a sub-county level. Our large sample of geocoded BMI data permitted a robust analysis across the state.

Data on height and weight were self-reported to the Oregon DMV and not verified by direct measurement. Though estimates do correlate well with those from the BRFSS, it is not possible to predict the error in any individual’s data [6]. By conducting analysis at the census tract level, uncertainty in the individual level estimates should have little effect on the regression results. The Oregon DMV data show associations between BMI and socioeconomic status consistent with other research, but without a better data to compare to we cannot fully assess how reporting bias varies between demographic groups or geographic areas. This information would be useful for correcting for self-report bias, but would require a large validation study using clinical height and weight measurements. We have seen no evidence of a systematic bias in height or weight reporting by county of residence, but if such a bias existed it could affect this study’s findings.

One major limitation is that eight years of DMV records were aggregated for this study. Aggregating eight years of data provided stable estimates for less populated areas, but also prevented us from comparing time trends, which could provide stronger evidence of causal effects. Though many people likely moved residences during that time, we do not know how many updated their address information on file with the DMV. Information on both current and former addresses may explain more of the observed variation in BMI. If higher BMIs are associated with lower incomes, and lower income families are moving rapidly to areas with fewer amenities, the relationship between community factors and BMI will be obscured. With a single snapshot of the DMV database, we were not able to ascertain whether people were more likely to update their address or their weight. Future studies may resolve this issue by tracking people through multiple years of DMV data.

Measures of the food and physical environments are based on a snapshot at a single point in time, but we compared them to DMV records issued in multiple years. With access to more data on the changing environment, sequential years of DMV records could be used for time series analysis. As public health practitioners build those datasets with community assessments, opportunities for future research grow.

People without driver licenses or state-issued ID cards are not included in this study. This may influence results, especially in agricultural areas where many undocumented workers live. Legislative efforts to issue driver licenses to undocumented workers may enhance data completeness for future studies. However, since nearly every adult resident in Oregon has a state-issued ID card, we consider the data to be representative of the state resident population [5]. 

This study used a simple ecological, cross-sectional design and is therefore susceptible to the ecological fallacy. We therefore urge caution when attributing causal effects. Using BMI data from individual DMV records for a multi-level or longitudinal analysis may yield stronger results. Many factors known to be associated with obesity, such as physical activity and dietary patterns, were not included in this study because reliable population level estimates are unavailable for areas smaller than counties. As more local data become available, this study may be replicated elsewhere with different results.

## 4. Conclusions

State-issued identification cards are a promising new data source for BMI surveillance and may offer new insight into the association between environment and weight status. Public health agencies should explore options for developing their own obesity estimates from identification card data. DMV data can reveal previously-unseen variations in weight status between small geographic areas, challenging some assumptions about drivers of obesity. With access to DMV data, more robust studies can be done on the associations between weight status, population characteristics, and the environment.
